# Supplementary material for: Melatonin Attenuates H2O2-Induced Oxidative Injury by Upregulating LncRNA NEAT1 in HT22 Hippocampal Cells
Source: Int J Mol Sci. 2022 Oct 25;23(21):12891. doi: 10.3390/ijms232112891 (PMC9657978; doi:10.3390/ijms232112891)
Supplement: Supplementary file 1 [file ijms-23-12891-s001.zip › ijms-1967640-supplementary/Supplementary Figure S1. The structure diagram of plasmid for establishing shRNA expression vector.pdf]

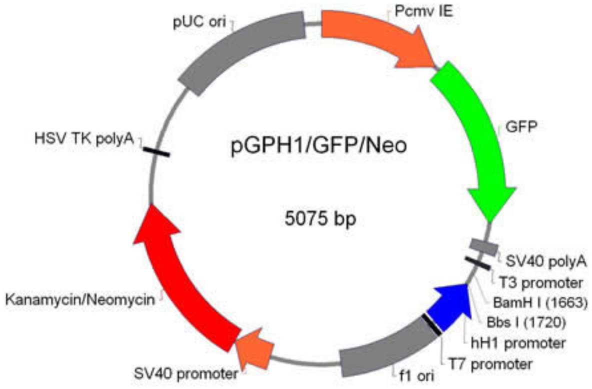

Figure S1. The structure diagram of plasmid for establishing shRNA expression vector (https://www.genepharma.com/show.php?ctype=0&coupid=562&cateid=112).
