# Supplementary material for: Melatonin Attenuates H2O2-Induced Oxidative Injury by Upregulating LncRNA NEAT1 in HT22 Hippocampal Cells
Source: Int J Mol Sci. 2022 Oct 25;23(21):12891. doi: 10.3390/ijms232112891 (PMC9657978; doi:10.3390/ijms232112891)
Supplement: Supplementary file 1 [file ijms-23-12891-s001.zip › ijms-1967640-supplementary/Supplementary Figure S2. Analysis of the correlation between 6 candidate lncRNAs and 806 differentially expressed mRNAs.pdf]

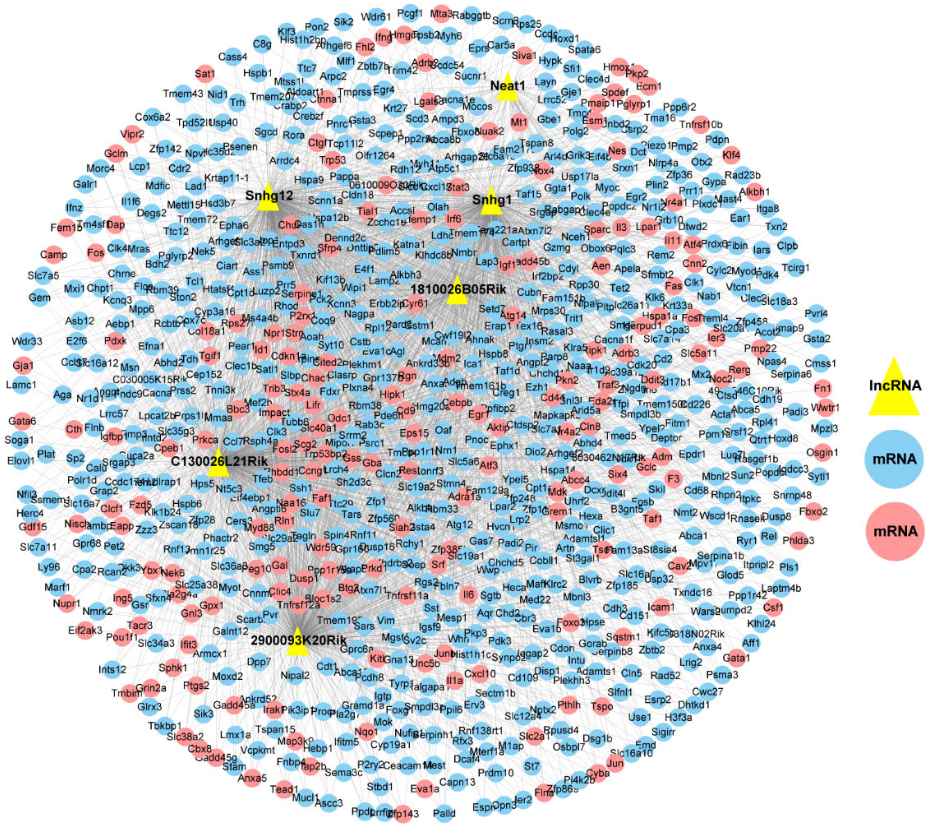

Figure S2. Analysis of the correlation between 6 candidate lncRNAs and 806 differentially expressed mRNAs. The triangle dots represent the 6 candidate lncRNAs, and the round dots represent 806 differentially expressed mRNAs which are significantly associated with the 6 lncRNAs. The red dots represent mRNAs related to aging, autophagy, apoptosis and oxidative stress, and the blue dots represent mRNAs that are related to other biological processes. Each line represents a correlation between two points.
