# Supplementary material for: Melatonin Attenuates H2O2-Induced Oxidative Injury by Upregulating LncRNA NEAT1 in HT22 Hippocampal Cells
Source: Int J Mol Sci. 2022 Oct 25;23(21):12891. doi: 10.3390/ijms232112891 (PMC9657978; doi:10.3390/ijms232112891)
Supplement: Supplementary file 1 [file ijms-23-12891-s001.zip › ijms-1967640-supplementary/Supplementary Table S2. The sequence of primer for qPCR.pdf]

Table S2. The sequence of primer for qPCR.

NEAT1	F	5′	GTTCCGTGCTTCCTCTTCTG	3′
	R	5′	CAGGGTGTCCTCCACCTTTA	3′
SNHG12	F	5′	TCGTTATGGCGCCTTGTAC	3′
	R	5′	CCATAACCCGTGCTTCCTT	3′
1810026B05Rik	F	5′	GCACCATAGACATCGAAGCA	3′
	R	5′	CCACTGTCCATTTGTGTTGG	3′
interferon-γ，*Ifn-γ*	F	5′	ACTGGCAAAAGGATGGTGAC	3′
	R	5′	TGTGGGTTGTTGACCTCAAA	3′
GATA binding protein 1，*Gata1*	F	5′	TTCTTCCCCCAAGTTTCATG	3′
	R	5′	TGCTGACAATCATTCGCTTC	3′
interleukin 1a，*Il1a*	F	5′	CCCGTCCTTAAAGCTGTCTG	3′
	R	5′	GAATCCAGGGGAAACACTGA	3′
heat shock protein 1a，*Hsp1a*	F	5′	TGGTGCTGACGAAGATGAAG	3′
	R	5′	AGGTCGAAGATGAGCACGTT	3′
eukaryotic translation initiation factor 2 alpha kinase 3，*Eif2ak3*	F	5′	GGTCTGGTTCCTTGGTTTCA	3′
	R	5′	TTCCCTCCAACCAGAACAAC	3′
solute carrier family 38 member 2，*Slc38a2*	F	5′	GCAGTACCCATCCTGACGTT	3′
	R	5′	ATGTCAGTCCCCACGATCTC	3′
activating transcription factor 4，*Atf4*	F	5′	TGCCCCTCTAGTCCAAGAGA	3′
	R	5′	AAGCAGCAGAGTCAGGCTTC	3′
death-associated protein，*Dap*	F	5′	GCAGTACCCATCCTGACGTT	3′
	R	5′	ATGTCAGTCCCCACGATCTC	3′
β-actin	F	5′	AGCCATGTACGTAGCCATCC	3′
	R	5′	GCTGTGGTGGTGAAGCTGTA	3′
